# Regulator of G protein signaling 2 is a key regulator of pancreatic *β-*cell mass and function

**DOI:** 10.1038/cddis.2016.216

**Published:** 2017-05-25

**Authors:** H Dong, Y Zhang, J Wang, D S Kim, H Wu, B Sjögren, W Gao, L Luttrell, H Wang

**Affiliations:** 1 Department of Surgery, Medical University of South Carolina, Charleston, SC 29425, USA; 2 College of Life Sciences, Qingdao Agricultural University, Qingdao 266109, PR China; 3 Tulane University School of Medicine, New Orleans, LA 70118, USA; 4 Department of Pharmacology and Toxicology, Michigan State University, East Lansing, MI 48824, USA; 5 Antagen Institute for Biomedical Research Inc., Boston, MA 02118, USA; 6 Department of Endocrinology, Medical University of South Carolina, Charleston, SC 29425, USA

## Abstract

Pancreatic *β*-cell death and dysfunction contributes to the pathogenesis of both type 1 and type 2 diabetes. We aimed to examine whether the regulator of G protein signaling protein 2 (RGS2), a multifunctional inhibitor of G protein-coupled receptor (GPCR) signaling, impacts *β*-cell death and function. Metabolic phenotypes, *β*-cell secretory function, and glucose and insulin tolerance were measured in RGS2 knockout (RGS2^−/−^) mice and their wild-type (RGS2^+/+^) littermate controls. *β*-Cell death was evaluated in RGS2-knockdown and -overexpressing *β* cells and RGS2^−/−^ islets by flow cytometry, western blot, ELISA, TUNEL staining, and apoptosis RT^2^ profiler PCR array analysis. *β*-Cell mass was evaluated in pancreases from RGS2^−/−^ and RGS2^+/+^ mice at 1 day, 4 weeks, and 25 weeks of age. Our data show that RGS2^−/−^ islets secreted more insulin than RGS2^+/+^ islets when challenged with glucose or exendin-4. RGS2-knockdown cells are susceptible to hypoxia induced cell death while RGS2-overexpressing cells are protected from cell death. Depletion of RGS2 in islets alters expression of apoptosis-related genes and RGS2^−/−^ islets are prone to apoptosis compared with RGS2^+/+^ islets. Ultimately, excessive insulin secretion and increased *β*-cell apoptosis contributed to a 70% reduction in pancreatic *β*-cell mass in RGS2^−/−^ mice compared with RGS2^+/+^ mice at 25 weeks of age. RGS2 has critical roles in maintaining pancreatic *β-*cell mass via modulating *β-*cell function and apoptosis. It may serve as a druggable target to help prevent pancreatic *β*-cell loss in the treatment of diabetes.

Stress-induced *β-*cell apoptosis reduces pancreatic *β*-cell mass and contributes to the development of diabetes. *β*-Cell mass is dynamic and regulated by various processes, including *β*-cell function/insulin secretion and apoptosis.^1^ *β* Cells are extremely vulnerable to stress-induced cell death.^2, 3^ For example, during the early phase of type 2 diabetes, the demand for increased insulin secretion to compensate for systemic insulin resistance eventually leads to impaired *β*-cell function and exhaustion, and gradually results in loss of *β-*cell mass through increased apoptosis.^4, 5, 6, 7^ Thus, identifying druggable targets that can be manipulated to regulate proper function and apoptosis of pancreatic *β* cells holds great promise for the treatment of diabetes.

Regulator of G protein signaling protein 2 (RGS2) is a multifunctional regulator of G protein signaling pathways. It interacts directly with G protein *α* subunits and accelerates GTP hydrolysis, leading to a more rapid termination of G protein signaling.^8^ RGS2 regulates vascular smooth muscle cell tone,^9^ impairs T-cell mediated immunity,^10, 11^ inhibits gastric inhibitory polypeptide (GIP)-stimulated cAMP production in embryonic kidney cells, and inhibits GIP-mediated insulin release in *β*TC3 insulinoma cells.^10^ RGS2^−/−^ mice manifest reduced T-cell proliferation and IL-2 production, increased anxiety response, decreased male aggression, exaggerated Angiotensin II-dependent hypertension, and reduced growth.^11, 12^ In addition to its function as a regulator of G protein-coupled receptor (GPCR) signaling, RGS2 has been shown to function as a stress adaptive protein by controlling ion channel currents, microtubule polymerization, and protein synthesis.^13^

We have previously observed that exposure of donor mice to carbon monoxide (CO) protects isolated islets from apoptosis after islet transplantation,^14^ an effect that correlates with upregulation of RGS2 expression at both mRNA and protein levels in isolated islets/*β* cells. However, the biological function of RGS2 in pancreatic *β* cells remains largely unknown. In this study, we aimed to determine the role played by RGS2 in *β*-cell apoptosis, using a systemic RGS2 knockout mouse model (RGS2^−/−^) and *β*TC3 cells in which the *RGS2* gene was knocked down by shRNA or overexpressed by lentiviral infection. Our data show that depletion of RGS2 leads to excessive insulin secretion and increased *β-*cell apoptosis, which contributes to reduced *β*-cell mass in aged mice. Together, these results suggest a pivotal role of RGS2 in maintaining pancreatic *β*-cell mass and function.

## Results

### RGS2^−/−^ islets secrete more insulin than wild-type islets

**Figure 1:**
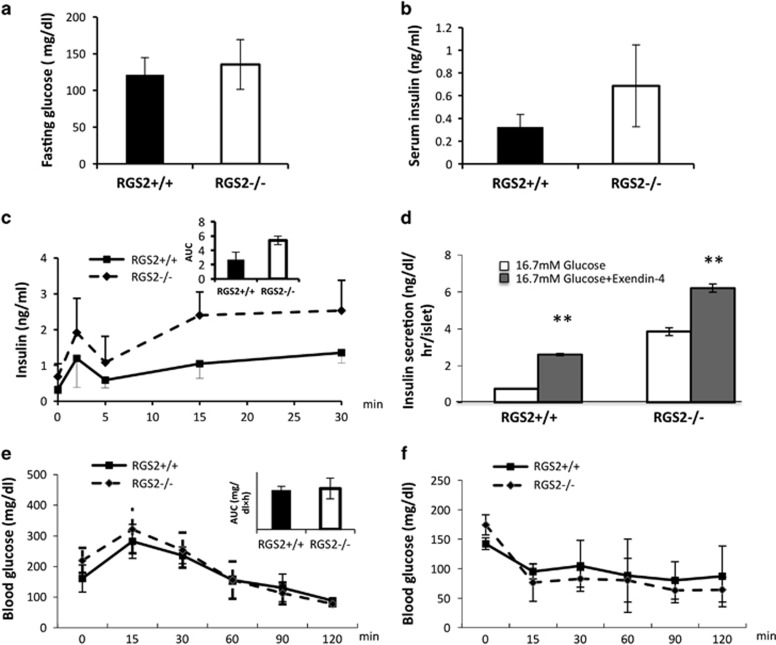
Glucose homeostasis in 8- to 10-week-old RGS2 knockout mice (RGS2^−/−^) and age-matched wild-type controls (RGS2^+/+^). (**a**) Fasting blood glucose levels; (**b**) serum insulin levels in male RGS2^+/+^ (RGS2^+/+^, *n*=6, black bar) and RGS2^−/−^ (RGS2^−/−^, *n*=6, white bar) mice. (**c**) Time course of serum insulin levels at 0, 2, 5, 15, and 30 min after glucose administration (3 g/kg, i.p., *n*=3–4 per group) and area under the curves (AUC, inset). (**d**) Insulin secretion in RGS2^−/−^ and RGS2^+/+^ islets after glucose (16.7 mM) with and without exendin-4 treatment. (**e**) Blood glucose levels after IPGTT in RGS2^−/−^ and RGS2^+/+^ mice and (inset) AUC. (**f**) ITT in RGS2^−/−^ and RGS2^+/+^ mice. For all panels, *n*=4 or 5 mice per group. Differences were compared by ANOVA, **P<0.01

We first examined whether depletion of the RGS2 gene produced a metabolic phenotype. On a C57BL/6 background, male RGS2^−/−^ mice showed similar body weights compared with age-matched RGS2^+/+^ mice at 8–10 weeks of age. No significant differences in serum glucose and insulin levels were observed (Figures 1a and b), suggesting no effect of RGS2 on glucose homeostasis.

To determine the role of RGS2 in pancreatic *β-*cell secretory function, we measured insulin release after intraperitoneal glucose stimulation (3 g/kg body weight, i.p.). No significant differences in insulin secretion were observed during the first phase (2 min) insulin secretion between RGS2^−/−^ mice and wild-type controls after glucose challenge. However, at 15 and 30 min after glucose injection, insulin secretion was significantly increased in RGS2^−/−^ mice (Figure 1c and inset). To confirm that depletion of RGS2 enhanced insulin secretion in *β* cells, glucose-stimulated insulin release was measured *ex vivo* in islets harvested from RGS2^−/−^ and wild-type mice in the presence or absence of the GLP-1 analog, Exendin-4. As is evident in Figure 1d, RGS2^−/−^ islets secreted significantly more insulin when exposed to 16.7 mM glucose, or Exendin-4, compared with islets from control mice. Thus, islets lacking RGS2 expression secrete more insulin than wild-type controls when challenged with glucose, suggesting that RGS2 serves as a negative regulator for insulin secretion.

To assess the impact of elevated insulin release on glucose disposal, we performed an intraperitoneal glucose tolerance test (IPGTT, 2 g/kg body weight) in RGS2^−/−^ and control mice. At 120 min after glucose challenge, there was no significant difference in either serum blood glucose level or glucose area under the curve between RGS2^−/−^ and RGS2^+/+^ mice (Figure 1e and inset). Results of an insulin tolerance test (ITT) showed that RGS2^−/−^ and control mice had similar blood glucose levels after insulin injection (0.75 U/kg), indicating similar insulin sensitivity (Figure 1f).

### RGS2 protects *β*TC3 cells from hypoxia stress-induced apoptosis

**Figure 2:**
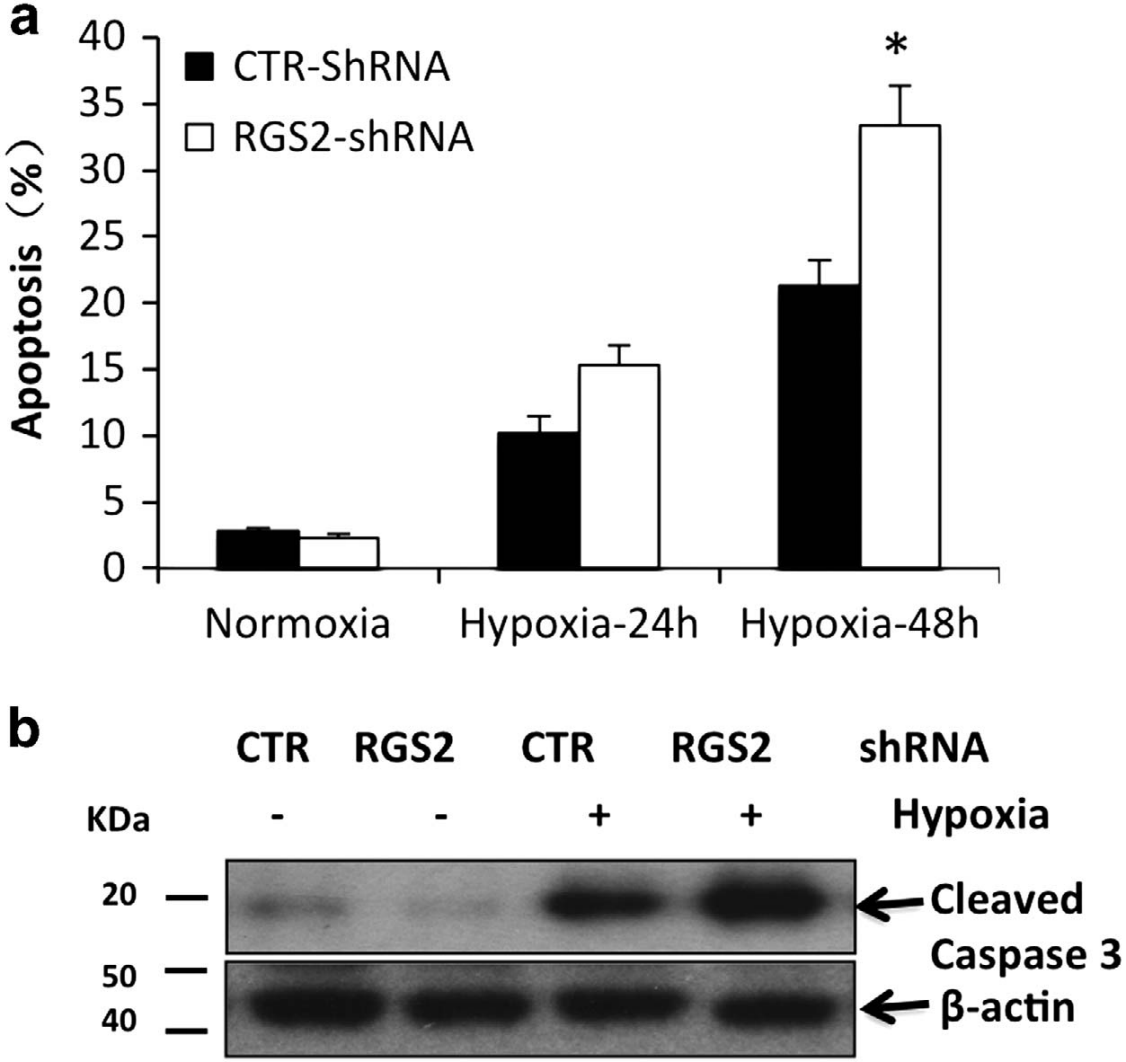
Knocking down of RGS2 expression by shRNA in *β* cells increases cell apoptosis. (**a**) Percentage of apoptotic cells in RGS2-knockdown (RGS2 shRNA) and control (control shRNA) cells cultured under normoxia (20% O_2_) and hypoxic (1% O_2_) conditions for 24 or 48 h (flow-cytometric analysis). (**b**) Protein expression of cleaved Caspase-3 and *β*-actin analyzed in *β*TC3 cells transfected with RGS2 shRNA or control shRNA (western blot analysis) at 8 h after hypoxia treatment. Data are representative from at least three individual experiments. *P<0.05, ANOVA test

To determine whether RGS2 has a role in *β*-cell apoptosis, we compared hypoxia-induced (1% O_2_) cell death in the presence or absence of RGS2 gene expression in an insulinoma cell line, *β*TC3 cells. RGS2 was knocked down using shRNA. Control cells were treated with control shRNA. As shown by flow-cytometry analysis, 10.2±1.8% of control cells underwent apoptosis, compared with 15.2±1.2% in RGS2-knockdown cells at 24 h after hypoxia (Figure 2a). At 48 h after hypoxia, 21.3±1.5% cells in control group underwent apoptosis, while 33.4±3.0% of cells were apoptotic in RGS2-knockdown cells (Figure 2a). Increased apoptosis was further confirmed by elevated levels of cleaved caspase-3 expression following 4 and 8 h of hypoxia in RGS2 knocking down cells compared with control cells as analyzed by western blot analysis (Figure 2b).

**Figure 3:**
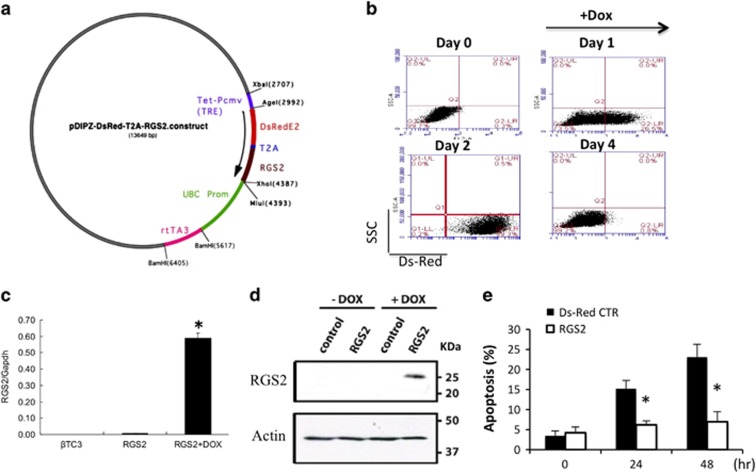
Overexpression of RGS2 in *β* cell protects cells from hypoxia-induced apoptosis. (**a**) Map of pDIPZ-DsRed-T2A-RGS2 lentiviral vector used for overexpressing RGS2 in *β*TC3 cells. (**b**) Flow-cytometry analysis of Ds-Red signaling before (day 0), 1 day after (day 1) and 2 days after (day 2) doxycycline (DOX) treatment. (**c**) Expression of RGS2 mRNA in RGS2-transfected cells in the presence or absence of DOX and in *β*TC3 control cells. (**d**) Protein expression of RGS2 analyzed by western blot in RGS2-transfected cells and control cells in the presence or absence of DOX. (**e**) Percentage of cell death in RGS2-overexpressing cells and control cells 24 and 48 h after hypoxia (1% O_2_) treatment (flow-cytometric analysis). Data are representative from at least three individual experiments. *P<0.05, Student's *t*-test

Next, we tested whether overexpression of RGS2 can protect *β*TC3 cells from apoptosis. RGS2 overexpression was achieved by infection with doxycycline (DOX)-inducible RGS2 lentivirus in which the RGS2 expression was reported by the Ds-Red fluorescence (Figures 3a and b). RGS2 overexpression upon DOX induction was confirmed by flow cytometry: Ds-Red+ cells were observed at day 1 after Dox induction. At 2 days after Dox induction, most cells were positive for Ds-Red. Furthermore, RGS2 overexpression was confirmed at mRNA (Figure 3c) and protein level (Figure 3d). When RGS2-overexpressing cells were challenged with hypoxia for 24 and 48 h, 7.1±1.2% and 8.2±1.9% cells underwent apoptosis compared with 15.3±2.3% and 23±3.4% of empty vector-infected control cells (Figure 3e), suggesting that RGS2 expression has a protective role in stress-induced *β*-cell apoptosis.

**Figure 4:**
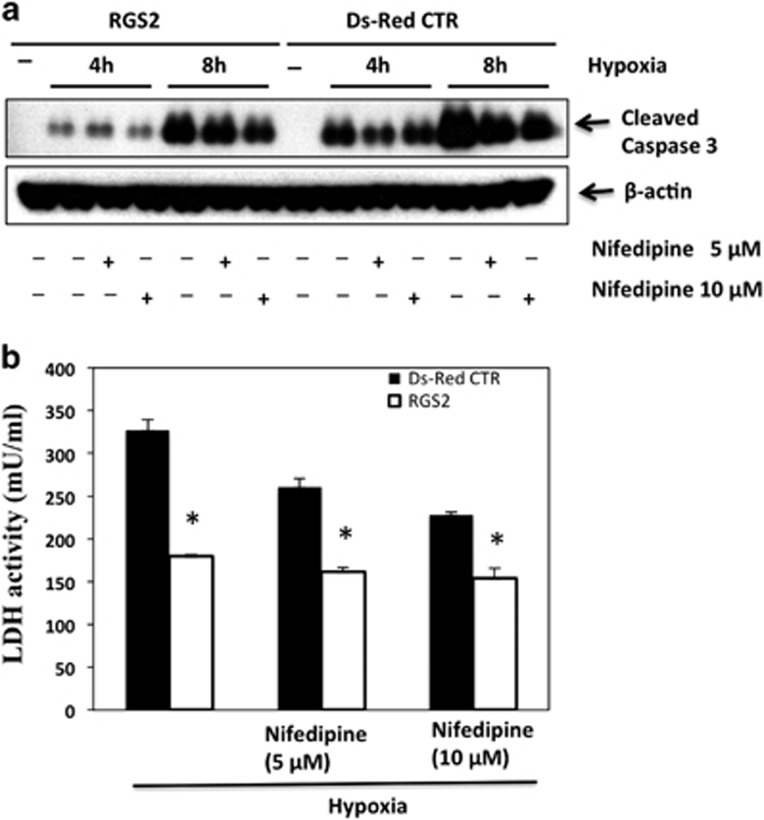
Inhibition of insulin secretion by calcium channel blocker protects *β*TC3 cells from hypoxia-induced cell death. (**a**) Expression of cleaved caspase-3 in RGS2-overexpressing (RGS2) or control (Ds-Red CTR) cells treated with vehicle or calcium channel blocker, nifedipine, at 5 or 10 *μ*M, and exposed to hypoxia for 4 or 8 h. (**b**) LDH activity in RGS2-overexpressing or control cells treated with vehicle or nifedipine and exposed to hypoxia for 24 h. *P<0.05, ANOVA test

To determine the impact of insulin secretion on RGS2-mediated *β*-cell survival, Ds-Red control or RGS2-overexpressing cells were treated with calcium channel blocker, nifedipine, at 5 or 10 *μ*M before exposure to hypoxia. Cell death was measured by expression of cleaved caspase-3 and lactate dehydrogenase (LDH) assay. In Ds-Red control cells, treatment with nifedipine dose-dependently inhibited hypoxia-induced *β*TC3 cell death (Figures 4a and b). In contrast, there was even less cell death in RGS2-overexpressing cells treated with vehicle compared with control cells treated with nifedipine. Treatment of RGS2-overexpressing cells with nifedipine at 10 *μ*M led to a slightly less cell death than those treated with vehicle, but the difference was not significant. These data indicate that RGS2 is important for *β-*cell survival and the protective effect was at least in part mediated by inhibition of insulin secretion.

### RGS2^−/−^ islets are more prone to cell death

**Figure 5:**
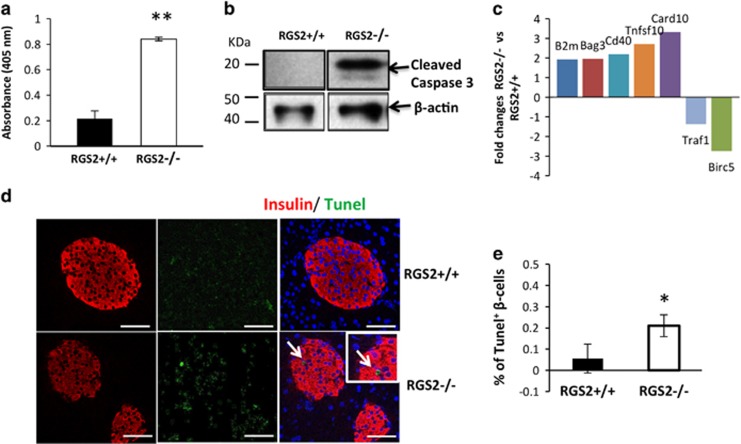
RGS2 is critical for *β-*cell apoptosis *in vivo*. (**a**) Absorbance at OD405 in RGS2^+/+^ and RGS2^−/−^ islets 24 h after hypoxia challenge (measured by the Cell Death Kit according to the manufacturer’s recommendation). (**b**) Protein expression of cleaved Caspase-3 and *β*-actin analyzed in isolated islets harvested from RGS2^+/+^ and RGS2^−/−^ mice after exposing to hypoxia for 6 h. (**c**) Transcriptional changes in expression of apoptosis-related genes 6 h after hypoxia challenge in islets from RGS2^+/+^ and RGS2^−/−^ mice (*n*=3–4). (**d**) Cell death in pancreas from RGS2^+/+^ and RGS2^−/−^ mice (TUNEL assay). Arrow points to TUNEL^+^ cell. Scale bar=50 *μ*M. (**e**) Percentage of TUNEL^+^ cells/Insulin^+^ cells (*n*=3 or 4 per group). *P<0.05, **P<0.01, Student's *t*-test

We also performed *ex vivo* studies to evaluate the role of RGS2 in pancreatic *β*-cell death. We found that islets harvested from RGS2^−/−^ mice at 8–10 weeks of age and challenged with hypoxia exhibited significantly more mono- and oligonucleosome production (reflecting DNA fragmentation/death) compared with control islets, as analyzed by a Cell Death Detection ELISA kit (Figure 5a) and cleaved caspase-3 expression (Figure 5b), suggesting that RGS2^−/−^ islets are more prone to hypoxia-induced cell death. We next performed a mouse apoptosis RT^2^ profiler PCR array to identify apoptosis-related genes that might be regulated by RGS2. Pro-apoptotic genes including B2m, Bag3, cd40, Tnfsf10 (TRAIL), and card10 were upregulated; whereas anti-apoptotic genes including Traf1 and survivin (Birc5) were downregulated in RGS2^−/−^ islets compared with wild-type controls (Figure 5c). These data suggest that RGS2 protects against hypoxia-induced *β* cell death by modulating the balance between expression of stress-induced death and survival signals.

The role of RGS2 in *β*-cell apoptosis was confirmed *in vivo*, where we measured islet cell death *in situ* in pancreas tissues from 8- to 10-week-old RGS2^+/+^ and RGS2^−/−^ mice using insulin and TUNEL co-staining. As shown in Figures 5d and e, increased numbers of apoptotic *β* cells (TUNEL^+^ insulin^+^ cells) were observed in pancreatic islets of RGS2^−/−^ mice compared with islets from RGS2^+/+^ mice. These data, again, confirm that RGS2 gene expression is critical for *β-*cell survival.

### RGS2 gene deletion accelerates age-related loss of pancreatic *β*-cell mass

**Figure 6:**
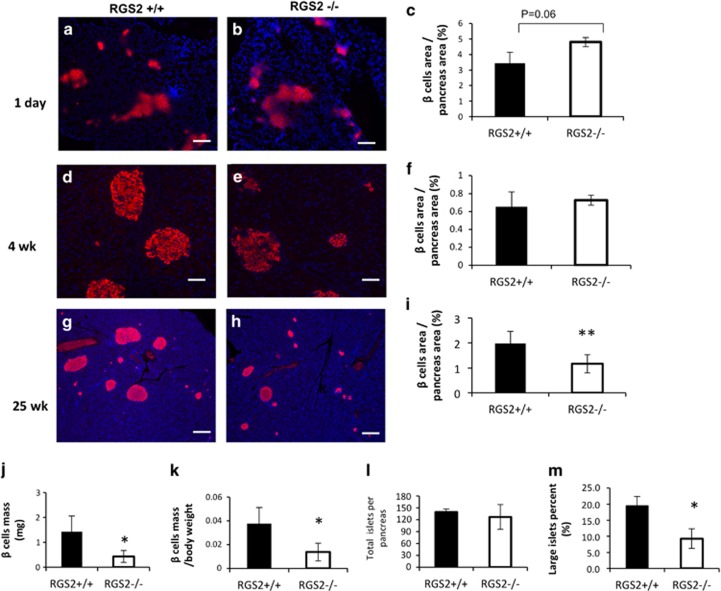
Comparison in pancreatic *β*-cell mass in RGS2^−/−^ and wild-type mice. (**a**) Representative micrographs of immunohistochemical staining of pancreatic islets from 1 -day-old (**a**) RGS2^+/+^ and (**b**) RGS2^−/−^ mice, from 4-week-old (**d**) RGS2^+/+^ and (**e**) RGS2^−/−^ mice, and from 25-week-old (**g**) RGS2^+/+^ and (**h**) RGS2^−/−^ mice. Red staining indicates insulin^+^ cells and blue staining indicates nuclei. Scale bar=50 *μ*M. *β*-Cell area per pancreas area at 1 day (**c**), 4 weeks (**f**), and 25 weeks (**i**) in RGS2^+/+^ and RGS2^−/−^ mice. *β*-Cell mass (**g**); *β*-cell mass per gram body weight (**k**); total number of islets per pancreas area (**i**) and percentage of large islets within a pancreas (**m**) at 25 weeks of age (*n*=4–5 per group). Differences were compared by Student’s *t*-test. *P<0.05, **P<0.01

**Figure 7:**
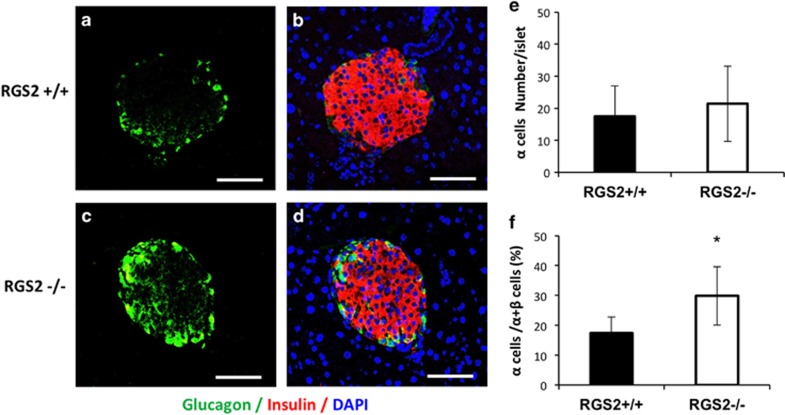
Characterization of *α* cells in pancreas. Representative micrographs of *β* cells (red) and *α* cells (green) in pancreatic tissue sections from RGS2^+/+^ (a and **b**) and RGS2^−/−^ (**c** and **d**) islets (identified by anti-insulin and anti-glucagon antibodies). Scale bar=100 *μ*M. (**e**) Percentages of *α* cells within an islet. (**f**) Percentage of *α* cells among *α* and *β* cells within pancreas. At least 50 islets have been counted in three different experiments. *P<0.05, Student’s *t*-test

As shown above, depletion of RGS2 led to excessive insulin secretion and increased apoptosis in 8- to 10-week-old mice. To determine whether accumulation of these effects contributes to a reduction in pancreatic *β*-cell mass *in vivo*, we measured pancreatic *β*-cell mass at 1 day, 4 weeks, and 25 weeks of age in RGS2^−/−^ and littermate control mice. No significant difference in *β*-cell mass was observed in mice at 1 day of age (Figures 6a–c) and 4 weeks of age (Figures 6d–f), suggesting that depletion of RGS2 had no impact on *β*-cell neogenesis during embryonic development and no impact on *β-*cell proliferation during pancreatic development after birth. However, by 25 weeks of age, the ratio of pancreatic *β* cell area to total pancreas area in RGS2^−/−^ mice was significantly reduced compared with wild-type controls (58.9% Figures 6g–i, *P*=0.01). *β*-Cell mass was also reduced by 70% (*P*=0.03) compared with wild-type controls (Figure 6j), and the ratio of *β-*cell mass to body weight was reduced by 63% (*P*=0.03) of controls (Figure 6k). The total number of pancreatic islets was not significantly different (Figure 6i). However, the size of RGS2^−/−^ islets was significantly reduced, that is, the percentage of large (>100 *μ*M in diameter) islets was 9.24±3.1%, compared with 19.4±2.9% in controls (Figure 6m). No difference in *α*-cell number per islet was observed (Figures 7a–e), but the percentage of *α* cells within an islet was 29.9±9.8% in RGS2^−/−^ mice compared with 17.4±5.5% in controls (*P*=0.03) (Figure 7f), reflecting the disproportionate loss of *β* cells and *α* cells.

**Figure 8:**
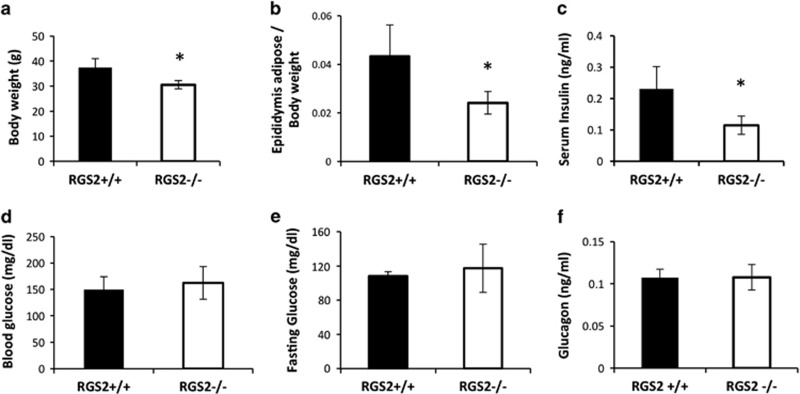
Metabolic phenotypes of RGS2^−/−^ and RGS2^+/+^ mice at 25 weeks of age. Body weights (**a**), ratio of epididymal adipose weight to body weight (**b**), serum insulin (**c**), fed (**d**) and fasting (**e**) insulin levels, and (**f**) glucagon levels in serum from 6-month-old RGS2^−/−^ and RGS2^+/+^ mice (*n*=4 or 5 per group). *P<0.05, Student’s *t*-test

As a next step, we characterized the metabolic phenotype of aged RGS2 mice. In contrast to 8- to 10-week-old mice, 25-week-old RGS2^−/−^ mice exhibited reduced body weight and reduced epididymis adipose weight compared with controls (Figures 8a and b). Serum insulin levels were also significantly reduced (0.23±0.07 ng/ml in RGS2^−/−^ *versus* 0.11±0.03 ng/ml in RGS2^+/+^ mice, *P*=0.01) (Figure 8c). However, no differences in fasting and non-fasting serum glucose or glucagon levels were observed between RGS2^−/−^ and RGS2^+/+^ mice (Figures 8d–f). Thus, while RGS2^−/−^ mice were able to maintain normoglycemia up to 6 months of age, loss of RGS2 appeared to lead to an age-related reduction in insulin secretory capacity, likely as a result of reduced *β*-cell mass.

## Discussion

In the present study, we found that RGS2 is a key regulator of pancreatic *β-*cell survival and is essential for retention of normal function and mass of *β* cells. We show that RGS2 is a negative regulator of glucose and exendin-4-induced insulin secretion. RGS2^−/−^ islets are more vulnerable to *β*-cell apoptosis *in vitro* and *in vivo*. Excessive insulin secretion and enhanced apoptosis contribute to a dramatic age-related loss of *β*-cell mass in RGS2^−/−^ mice.

*β-*Cell mass and function are controlled by glucose and hormones/neurotransmitters that activate GPCRs or receptor tyrosine kinases.^15^ Incretins acting on GPCRs, including glucose-dependent GIP and GLP-1, synergistically promote glucose-stimulated insulin secretion (GSIS). Components of G protein signaling networks, including GLP-1 receptor,^16^ G protein-coupled receptor 119,^17^ as well as Gs*α* ^18^ and the Gs*α* mediated signaling,^19^ have important roles in regulating *β-*cell mass. As a regulator of GPCR signaling, RGS2 inhibits GIP-induced cAMP responses and insulin secretion.^10, 16, 17, 18^

*β*-Cell exhaustion after excessive insulin secretion leads to *β*-cell death.^20^ Here, we found that RGS2 functions as a negative regulator for glucose, and exendin-4-induced insulin release and RGS2 deletion led to increased insulin secretion in islets. Excessive insulin secretion in RGS2^−/−^ islets due to persistent augmentation of G protein signaling may in part contribute to *β-*cell exhaustion and apoptosis leading to reduced *β*-cell mass.

RGS2 is a negative regulator for Gq/11 signaling,^8^ as well as for Gi and can also regulate Gs- and Gi-coupled *β*2-adrenoceptors in a cell- and context-specific manner.^21, 22^ However, the metabolic phenotypes of RGS2^−/−^ mice were substantially different from the phenotypes of *β* cell-specific G*α*q/G*α*11-deficient mice, which showed no differences in pancreatic *β*-cell mass, number, or histology, but exhibited hyperglycemia and a decrease in early insulin secretion.^23^ In contrast, *β* cell-specific Gs*α* conditional knockout mice were similar to RGS2^−/−^ mice in that they exhibited reduced average islet size, reduced *β*-cell mass, and increased *β-*cell apoptosis. These mice differed from RGS2^−/−^ mice in that they had severe hyperglycemia, glucose intolerance, hypoinsulinemia, reduced islet insulin content, and glucose-stimulated insulin release.^18^ It is also worth noting that the impact of G*α*_s_ deletion on *β*-cell mass and proliferation was observed at birth, but only later in RGS2^−/−^ mice, suggesting distinct functions of RGS2 and Gs*α* in pancreatic *β* cells.

RGS2 has been suggested to be a stress responsive gene that suppresses protein synthesis after stress.^25^ The fact that RGS2 can be induced by CO (Wang, *unpublished data*), which has a protective role when exposed to islet donors before islet harvest, is consistent with a potential role as a stress responsive gene. In addition, our results indicate that RGS2 is a negative regulator of GLP-1-mediated insulin release. It is not clear, however, whether RGS2 mediates the pro-proliferative or anti-apoptotic functions of GLP-1 as previously suggested,^26^ or *vice versa*. Nonetheless, it is clear that in addition to its conventional role as a regulator of G protein signaling, RGS2 has profound GPCR-independent functions in pancreatic *β* cells.

The RGS2^−/−^ mice used in our study were global knockout mice with defects in multiple organs.^11^ Therefore, we cannot exclude potential contributions of systemic stress to the reduction of pancreatic *β*-cell mass in these mice. Nevertheless, our confirmation that RGS2 negatively regulates insulin secretion and exhibits anti-apoptotic properties in islets and *β*TC3 cells supports a critical role of RGS2 in *β* cells.

In conclusion, we found that *β-*cell dysfunction and increased apoptosis in RGS2^−/−^ islets/*β* cells contribute to reduced *β-*cell mass in RGS2^−/−^ mice. Elucidation of the roles of RGS2 in regulating pancreatic *β-*cell mass will likely lead to a better understanding and, potentially, to novel therapies for treatment of diabetes.

## Materials and Methods

### Mice

Heterozygous RGS2 (RGS2^+/−^) mice on the C57BL/6 background were a generous gift from Dr. Ken Blumer (Washington University, School of Medicine, St. Louis, MO, USA). RGS2 knockout (RGS2^−/−^) and wild-type control (RGS2^+/+^) mice were generated by mating of RGS2^+/−^ mice as described.^11^ To be consistent, only male mice were used in our study. Animals were maintained under a standard 12 h :12 h light-dark cycle. Food and water were available *ad libitum*. All experiments were approved by the Institutional Animal Care and Use Committee at the Medical University of South Carolina.

### Glucose and insulin tolerance tests

For IPGTT, mice that had been fasted overnight received an i.p. injection of 2 mg/g body weight glucose. For insulin tolerance test, mice that had been fasted for 5 h received an i.p. injection of recombinant human insulin (0.75 U/kg; Humulin, Eli Lily, Indianapolis, IN, USA). Blood samples were taken from the tail vein before (0 min) and 15, 30, 60, 90, and 120 min after glucose or insulin injection. Serum glucose levels were determined using an ACCU-CHEK glucometer (LifeScan, Mountain View, CA, USA). Area under curves were calculated using standard methods.

### Glucose-stimulated insulin secretion

Mice were fasted overnight and then injected with d-glucose (3 mg/g). Blood was collected from the tail vein at 0, 2, 5, 15, and 30 min post glucose challenge. Serum insulin was measured using a Mouse Chemiluminescent Insulin ELISA kit (ALPCO, Salem, NH, USA).

### Deletion or overexpression of RGS2 in *β*TC3 cells

RGS2 was depleted in *β*TC3 cells by transfection with vector encoding shRNA for RGS2 (Santa Cruz Biotechnology, Santa Cruz, CA, USA). The Tet-On inducible pTIPZ lentiviral vector (Clontech Laboratories, Mountain View, CA, USA) was used to generate lentiviral vector that can used to induce RGS2 expression. The red fluorescence protein (Ds-Red) was inserted downstream of the Tet-PCMV promoter and was used as an empty vector control. The RGS2 cDNA (Open Biosystems, Lafayette, CO, USA) was inserted downstream of the Ds-Red gene that is separated by the T2A sequence to generate a pDIPZ-DsRed-T2A-RGS2 vector. Expression of RGS2 was induced by treatment with DOX at 600 ng/ml, and was detected by Ds-Red fluorescence.

### Cell death analysis

For detection of islet/*β*-cell death *in vitro*, cells were exposed to hypoxia (1% O_2_) for 24 or 48 h. Cells were harvested and washed with PBS containing 5 mM EDTA. After fixation in 70% ethanol at 4 °C for 2 h, cells were collected, re-suspended in PBS containing 250 *μ*g/ml RNase A, and incubated at 37 °C for 30 min. After propidium iodide staining, 2 × 10^5^ cells per sample were analyzed by flow cytometry. The portion of the apoptotic cells was calculated according to the apoptotic peak in the cell distribution against the propidium iodide fluorescence per cell. Cell death was also measured using a Cell Death Detection ELISA Kit and the colorimetric assay in medium using a LDH cytotoxicity detection kit (Clontech) as suggested by manufacturer (Roche, Indianapolis, IN, USA). For detection of *β*-cell apoptosis inside the pancreas, pancreatic tissue sections were incubated with terminal deoxynucleotidyl transferase in the presence of Biotin-dUTP (Roche Diagnostics). TUNEL-positive cells were revealed with Alexa Fluor 488-Streptavidin. Sections were double stained for insulin to distinguish *β* cells. Images were obtained by confocal microscopy. The numbers and fraction of TUNEL^+^ cells within an islet were calculated.

### Real-time RT-PCR analysis for gene expression

Total RNA was extracted from islets/*β* cells using an RNeasy kit (Qiagen, Valencia, CA, USA). Expression of apoptosis-related genes was analyzed using a mouse apoptosis RT^2^ profiler PCR array that profiles expression of 84 key genes involved in programmed cell death according to the manufacturer’s recommendation (Qiagen).

### SDS-PAGE and western blot

Protein concentration in the lysates was determined using the BCA assay (Pierce, Rockford, IL, USA) and adjusted with an appropriate volume of Laemmli buffer (Bio-Rad, Hercules, CA, USA). Equal amounts of protein were resolved on a 12% SDS-PAGE gel for 1 h at 160 V. Samples were transferred to an Immobilon-P membrane (Millipore, Billerica, MA, USA) for 1 h at 100 V, 400 mA on ice, and subjected to western blot analysis. The membrane was blocked with Tris-buffered saline, 5% (w/v) non-fat dry milk for 30 min and probed overnight at 4 °C with a rabbit RGS2 antibody (a gift from Dr. David Siderovski). The membrane was probed for 1 h with horseradish peroxidase (HRP)-conjugated Goat anti-rabbit (A0545; 1 : 10 000, Sigma-Aldrich, St. Louis, MO, USA) secondary antibody diluted in TBS-T, 5% (w/v) non-fat dry milk. The protein bands were visualized on autoradiography film using the Super Signal West Pico chemiluminescent substrate (Pierce, Waltham, MA, USA).

### Quantification of pancreatic *β*-cell mass

Whole-mouse pancreases were embedded in paraffin and cryosections prepared as described.^24^ Consecutive sections 100 *μ*m apart spanning the entire pancreas were collected and stained with guinea pig anti-insulin antibody and rabbit anti-glucagon antibodies (Dako, Carpinteria, CA, USA). Digital images of each section were obtained using a Carl Zeiss M2 microscope (Carl Zeiss, Oberkochen, Germany). Total tissue area and *β*-cell and *α-*cell areas were determined using Image-Pro Plus software (Media Cybernetics, Rockville, MD, USA) by quantification of the cross-sectional *β*-cell or *α*-cell area divided by the cross-sectional area of total tissue. For islet size and *β*-cell area quantification, the whole area for all sections was imaged. The total insulin-positive (*β* cell) area and the average islet size (calculated from individual islet diameter) were calculated from these data for each mouse.

### Statistical analysis

Data are expressed as mean±standard deviation. Differences between groups were compared for statistical significance by Student’s *t-*test and one-way ANOVA with Bonferroni correction; *P*<0.05 was considered as significant.

## Abbreviations

RGS2: regulator of G protein signaling 2
GPCR: G protein-coupled receptor
GLP-1: glucagon-like peptide-1
B2m: Pro-apoptotic genes including *β*2-microglobulin
Bag3: Bcl2-associated athanogene 3
Card10: caspase recruitment domain family member *10*
Traf1: TNF receptor-associated factor 1
GSIS: glucose-stimulated insulin secretion
IPGTT: intraperitoneal glucose tolerance test
ITT: insulin tolerance test
CO: carbon monoxide
DOX: doxycycline
GIP: gastric inhibitory polypeptide
LDH: lactate dehydrogenase
